# The HPV Vaccine Controversy. Sex, Cancer, God, and Politics: A Guide for Parents, Women, Men, and Teenagers

**DOI:** 10.1038/sj.bjc.6605643

**Published:** 2010-04-13

**Authors:** S Franceschi

**Affiliations:** 1 International Agency for Research on Cancer, 150 Cours Albert Thomas, 69372, Lyon Cedex 08, France

In June 2006, the United States Centers for Disease Control first recommended the administration of the human papillomavirus (HPV) vaccine to 11–12-year-old girls. *The HPV Vaccine Controversy* is a testimony of the amazing uprise of interest and discussion that accompanied this recommendation and the arrival of the first HPV vaccine, and follows it through 2008, up to the time when the book was published. The author, Dr Shobha Krishnan, explains that the book's target audience is parents, women, men and teenagers.

Overall, Dr Krishnan provides a well-balanced view of the strengths and weaknesses of the currently available prophylactic HPV vaccines, including the need to continue screening efforts, and to avoid feelings of fear and guilt on the part of a woman when a HPV infection is detected during cervical cancer screening. The book, however, is very long and is full of technicalities. It made me wonder if, in order to make up their mind, the lay public was anxious to know so many details on HPV types that are not included in the current vaccines, or on the cervical cancer staging system. It is true that when ‘…sex, science, and morals of society intersect.’ (author's note, page xiii), everybody has something to say, but other lines of reflection could have been, in my view, more fecund for the lay public.

How was it, for instance, that after 13 years of deliberation, both Merck and Glaxo Smith Kline signed royalty agreements with four, mainly tax-payer-supported, institutes (pages 97–98) so that their original research could be applied to developing and manufacturing ‘a vaccine that was released at the highest price in history’? Why is there so much insistence on sexually transmitted diseases when the HPV vaccine has been conceived, and should have been promoted, as the vaccine against cervical cancer?

An obvious side effect of the length of the book is the increase in the scope for inaccuracies and already out-of-date scientific statements. For instance, some myths are presented as facts, for example, ‘The teenage cervix is most vulnerable to HPV…’ (page 23), or ‘Men should be equal players in reducing the viral load in society’ (page 134). The first statement is often heard, but it has no clear scientific support. The second is the result of a misunderstanding (although it is a general wish that a cheap vaccine may be ultimately given to all infants, regardless of sex). In reality, good vaccine coverage of one sex is enough to curb HPV infection for the same reason Dr Krishnan believes the contrary, that is, because transmission is mainly from one sex to another. Women, however, have a stronger need to be vaccinated than men as it is they who develop the most severe and frequent disease associated with HPV, that is, cervical cancer.

Appropriately, the book ends not in the United States, but in India, with a touching expression of gratitude from a 37-year-old woman whose life was saved in the 1990 s by the early detection of cervical cancer (pages 187–188). Almost certainly, her daughters have already missed the chance to benefit from the vaccine against cervical cancer. Will her granddaughters miss it too?

